# Variable transformation of singular cylindrical vector beams using anisotropic crystals

**DOI:** 10.1038/s41598-020-62546-2

**Published:** 2020-03-27

**Authors:** Svetlana N. Khonina, Alexey P. Porfirev, Nikolay L. Kazanskiy

**Affiliations:** 1 Samara National Research University, Samara, 443086 Russia; 2 Image Processing Systems Institute of RAS - Branch of the Federal Scientific Research Centre “Crystallography and Photonics” of Russian Academy of Sciences, Samara, 443001 Russia

**Keywords:** Optics and photonics, Applied optics, Optical techniques

## Abstract

We demonstrated and investigated, both theoretically and experimentally, the transformation of cylindrical vector beams with an embedded phase singularity under the condition of focusing perpendicularly to the axis of the anisotropic calcite crystal. Theoretical and numerical analysis, performed on the basis of decomposing the light field into a set of plane waves for an anisotropic medium, allowed us to study the dependence of the structural transformation of the initial laser beam on the polarisation and phase state in detail. The proposed approach allows one to perform the visual recognition of cylindrically-polarised vector beams of various orders and can be used for the demultiplexing of information channels in the case of polarisation-division multiplexing. The experimentally-obtained results agree with the theoretical findings and demonstrate the reliability of the approach.

## Introduction

Nonparaxial propagation of laser modes in a medium with strong anisotropy leads to complex polarisation-phase transformations^1–3^, which allow for the formation of inhomogeneously polarised beams^4–9^, as well as beams with vortex phase singularities^10–13^. In the latter case, it is possible to observe the transformation of the initial circularly-polarised beam with non-zero spin angular momentum into a laser beam with non-zero orbital angular momentum. These effects can also be produced by focusing laser radiation along the axis of an anisotropic crystal, due to the interaction of the ordinary and extraordinary beams^14^.

When laser beams propagate perpendicularly to the crystal axis^2,3,15–22^, various effects are observed. In particular, for Bessel beams, pronounced astigmatic distortion of the ring structure of the beam occurs^3,18,21,22^. For Gaussian beams, the astigmatic transformation is hardly noticeable^23^, since natural crystals, as a rule, have a slight relative difference between the ordinary and extraordinary refractive indices. To enhance the astigmatic transformation and make it visually noticeable, sharply focused vortex Gaussian beams are used. In this case, the influence of the polarisation of the illuminating beam becomes especially important^24^. In the present paper, the variable transformation of singular cylindrical vector beams (CVBs)^25–27^ using an anisotropic crystal is investigated theoretically, numerically, and experimentally. In order to introduce into the initial CVB a complex singular phase of superposition of optical vortices, we used a spatial light modulator (SLM). Earlier, SLMs were also used for modification of incident laser radiation and the generation of amplitude-squeezed high-order vector beams by means of a collinear interferometric technique^28^. Because of the use of the SLM, the vortex composition and the weight ratio in this superposition can change dynamically. Following this, when the generated field is focused perpendicularly to the axis of an anisotropic crystal, a selective (polarisation-dependent) astigmatic transformation of the individual components of the electric field occurs. This phenomenon has been studied previously in uniformly-polarised vortex beams^24^. In this paper, the more general case of cylindrically-polarised singular beams is considered. CVBs, including high orders, are of practical interest in such areas as compressed optical data transfer^29^, amplitude-polarisation modulation of focal distributions^30–32^, sharp focusing^33–37^, and others. The combination of CVBs with a singular phase, on the one hand, provides an additional degree of freedom of the formed distributions^34–38^, but on the other hand, their interaction leads to unexpected effects, including polarisation transformations^39–41^. These effects require additional research, especially when focusing inside an anisotropic medium.

## Results

### Theoretical analysis

We start by analysing the nonparaxial propagation of an electromagnetic wave perpendicular to the crystal axis using the plane wave decomposition method^42^ for an anisotropic medium^43^. If the *c*-axis of the crystal is directed along the coordinate axis *Y*, then the dielectric permittivity tensor is as follows (in the absence of charges, we assume the magnetic permeability *μ* is equal to 1):

1 $$\overleftrightarrow{{\varepsilon }_{y}}=(\begin{array}{ccc}{\varepsilon }_{0} & 0 & 0\\ 0 & {\varepsilon }_{1} & 0\\ 0 & 0 & {\varepsilon }_{0}\end{array}),$$

where *ε*_0_ and *ε*_1_ are the ordinary and extraordinary dielectric constants, respectively, of the uniaxial crystal.

The electric component of the electromagnetic wave propagating along the optical axis *z* has the following form^43^:

2 $$\begin{array}{rcl}{\bf{E}}(u,v,z) & = & \mathop{\iint }\limits_{{\alpha }^{2}+{\beta }^{2}\le 1}\left\{{c}_{0}(\alpha ,\beta ){{\bf{e}}}_{0}(\alpha ,\beta )\exp \left[ik{\gamma }_{0}(\alpha ,\beta )z\right]\right.\\  &  & +\ {c}_{1}(\alpha ,\beta ){{\bf{e}}}_{1}(\alpha ,\beta )\exp \left[ik{\gamma }_{1}(\alpha ,\beta )z\right]\\  &  & \times \ \exp \left[ik(\alpha u+\beta v)\right]d\alpha d\beta ,\end{array}$$

where (*u*, *v*) are the Cartesian coordinates at the distance *z* from the initial plane defined in the Cartesian coordinates (*x*, *y*), **e**_0_(*α*, *β*) and **e**_1_(*α*, *β*) are the eigenvectors of the ordinary and extraordinary beams, respectively. With regard to the tensor described by Eq. (1), it has the following form:

3 $${{\bf{e}}}_{0}(\alpha ,\beta )=\left(\begin{array}{c}{e}_{0x}(\alpha ,\beta )\\ {e}_{0y}(\alpha ,\beta )\\ {e}_{0z}(\alpha ,\beta )\end{array}\right)=\left(\begin{array}{c}1\\ 0\\ -\alpha /{\gamma }_{0}(\alpha ,\beta )\end{array}\right),{{\bf{e}}}_{1}(\alpha ,\beta )=\left(\begin{array}{c}{e}_{1x}(\alpha ,\beta )\\ {e}_{1y}(\alpha ,\beta )\\ {e}_{1z}(\alpha ,\beta )\end{array}\right)=\left(\begin{array}{c}\alpha \beta \\ {\beta }^{2}-{\varepsilon }_{0}\\ -\beta /{\gamma }_{1}(\alpha ,\beta )\end{array}\right),$$

and the eigenvalues are defined by the following expressions:

4 $$\begin{array}{rcl}{\gamma }_{0}(\alpha ,\beta ) & = & \sqrt{{\varepsilon }_{0}-{\alpha }^{2}-{\beta }^{2}},\\ {\gamma }_{1}(\alpha ,\beta ) & = & \sqrt{{\varepsilon }_{1}-{\alpha }^{2}-\frac{{\varepsilon }_{1}}{{\varepsilon }_{0}}{\beta }^{2}}.\end{array}$$

The coefficients in Eq. (2):

5 $$\begin{array}{rcl}{c}_{0}(\alpha ,\beta ) & = & {\Delta }^{-1}\left[{S}_{x}(\alpha ,\beta ){e}_{1y}(\alpha ,\beta )-{S}_{y}(\alpha ,\beta ){e}_{1x}(\alpha ,\beta )\right],\\ {c}_{1}(\alpha ,\beta ) & = & {\Delta }^{-1}\left[{S}_{y}(\alpha ,\beta ){e}_{0x}(\alpha ,\beta )-{S}_{x}(\alpha ,\beta ){e}_{0y}(\alpha ,\beta )\right],\\ \Delta  & = & {e}_{0x}(\alpha ,\beta ){e}_{1y}(\alpha ,\beta )-{e}_{0y}(\alpha ,\beta ){e}_{1x}(\alpha ,\beta ),\end{array}$$

where *S*_*x*,*y*_(*α*, *β*) are the components of the spatial spectrum for the transverse components *E*_*x*,*y*_(*x*, *y*, 0) of the input electric field:

6 $$\left(\begin{array}{c}{S}_{x}(\alpha ,\beta )\\ {S}_{y}(\alpha ,\beta )\end{array}\right)=\frac{1}{{\lambda }^{2}}\mathop{\iint }\limits_{\Omega }\left(\begin{array}{c}{E}_{x}(x,y,0)\\ {E}_{y}(x,y,0)\end{array}\right)\exp \left[-ik(\alpha x+\beta y)\right]dxdy,$$

where Ω is the domain of the input field definition.

After substituting Eqs. (3)–(5) into Eq. (2), we obtain the expressions for the individual field components in the following form:

7 $$\begin{array}{rcl}{E}_{x}(u,v,z) & = & \mathop{\iint }\limits_{{\alpha }^{2}+{\beta }^{2}\le 1}\left\{{S}_{x}(\alpha ,\beta )\exp \left[ik{\gamma }_{0}(\alpha ,\beta )z\right]\right.\\  &  & +\ \frac{{S}_{y}(\alpha ,\beta )\alpha \beta }{({\beta }^{2}-{\varepsilon }_{0})}\left.\left\{\exp \left[ik{\gamma }_{1}(\alpha ,\beta )z\right]-\exp \left[ik{\gamma }_{0}(\alpha ,\beta )z\right]\right\}\right\}\\  &  & \times \ \exp \left[ik(\alpha u+\beta v)\right]d\alpha d\beta ,\end{array}$$

8 $${E}_{y}(u,v,z)=\mathop{\iint }\limits_{{\alpha }^{2}+{\beta }^{2}\le {\sigma }^{2}}{S}_{y}(\alpha ,\beta )\exp \left[ik{\gamma }_{1}(\alpha ,\beta )z\right]\exp \left[ik(\alpha u+\beta v)\right]d\alpha d\beta ,$$

9 $$\begin{array}{rcl}{E}_{z}(u,v,z) & = & \mathop{\iint }\limits_{{\alpha }^{2}+{\beta }^{2}\le 1}\left\{-\frac{{S}_{x}(\alpha ,\beta )\alpha }{{\gamma }_{0}(\alpha ,\beta )}+\frac{{S}_{y}(\alpha ,\beta )\beta }{\left({\beta }^{2}-{\varepsilon }_{0}\right)}\left\{\frac{{\alpha }^{2}}{{\gamma }_{0}(\alpha ,\beta )}\exp \left[ik{\gamma }_{0}(\alpha ,\beta )z+\ {\gamma }_{1}(\alpha ,\beta )\exp \left[ik{\gamma }_{1}(\alpha ,\beta )z\right]\right]\right\}\right\}\\  &  & \times \ \exp \left[ik(\alpha u+\beta v)\right]d\alpha d\beta .\end{array}$$

The contribution of the *y*-component of the original field in the integral in Eq. (7) (the second term in the integral) is small: this term disappears in an isotropic medium (that is, when *γ*_1_(*α*, *β*) = *γ*_0_(*α*, *β*)). Thus, when the laser beam passes through a crystal whose axis is directed along the coordinate axis *Y*, the *x*-component of the field is not significantly distorted, propagating as in an isotropic medium. This effect was noted earlier in^21,22,24^. The integral in Eq. (8) contains only the *y*-component of the original field, which undergoes an astigmatic transformation due to its complex form. A detailed analysis of this effect was carried out earlier for the Bessel^21^ and Gaussian beams^24^.

As follows from Eq. (9), the most complex transformation occurs with the longitudinal component of the electric field. However, it is not visually noticeable, since the energy of the longitudinal component is insignificant compared to the contribution of the transverse components even for tight focusing^8,23^. Similar results were obtained previously for beams with homogeneous polarisation based on the Rayleigh-Sommerfeld vector integrals for an anisotropic medium^24,43^.

Next, we consider the more general case of cylindrically-polarised beams with a singular phase. For this, in addition to Eqs. (7)–(9), it is necessary to consider the components of the spatial spectrum in Eq. (6). The field in Eq. (6) with cylindrical polarisation of *p*-th order is described as follows^34–37,44,45^ (we will call these polarisations *p*-th order radial and azimuthal polarisation, respectively):

10 $$\begin{array}{rcl}{{\bf{E}}}_{\perp }^{Rad,p}(x,y,0) & = & \left(\begin{array}{c}\cos (p\phi )\\ \sin (p\phi )\end{array}\right){E}_{0}(x,y),\\ {{\bf{E}}}_{\perp }^{Az,p}(x,y,0) & = & \left(\begin{array}{c}-\sin (p\phi )\\ \cos (p\phi )\end{array}\right){E}_{0}(x,y),\end{array}$$

where *ϕ* is the polar angle.

It can be seen from Eq. (10) that the radial and azimuthal polarisations are similar up to a rotation of 90 degrees or the *x*- and *y*-components interchanging; therefore, we further analyse only the radial polarisation in the polar coordinates. We also consider the presence of a singular phase in the laser beam in the form of a superposition of *Q* optical vortices with different *m*_*q*_-th orders:

11 $${{\bf{E}}}_{\perp }^{Rad,p}(r,\phi ,0)={E}_{0}(r)\left(\begin{array}{c}\cos (p\phi )\\ \sin (p\phi )\end{array}\right)\mathop{\sum }\limits_{q=1}^{Q}{c}_{q}\exp (i{m}_{q}\phi ),$$

where *E*_0_(*r*) is an arbitrary axisymmetric field corresponding to the illuminating beam.

Then, writing Eq. (6) in polar coordinates and substituting Eq. (11) into it, the following expression is obtained:

12 $$\left(\begin{array}{c}{S}_{x}(\sigma ,\theta )\\ {S}_{y}(\sigma ,\theta )\end{array}\right)=\frac{\pi }{{\lambda }^{2}}\mathop{\sum }\limits_{q=1}^{Q}{c}_{q}\left(\begin{array}{c}\exp \left[i({m}_{q}+p)\theta \right]{T}_{{m}_{q}+p}(\sigma )+\exp \left[i({m}_{q}-p)\theta \right]{T}_{{m}_{q}-p}(\sigma )\\ -i\exp \left[i({m}_{q}+p)\theta \right]{T}_{{m}_{q}+p}(\sigma )+i\exp \left[i({m}_{q}-p)\theta \right]{T}_{{m}_{q}-p}(\sigma )\end{array}\right),$$

where ${T}_{q}(\sigma )={i}^{q}{\int }_{0}^{R}{E}_{0}(r){J}_{q}(kr\sigma )rdr,\sigma =\sqrt{{\alpha }^{2}+{\beta }^{2}}$. It is clear that the presence of a vortex singularity in the beam leads to a change in the polarisation state of the beam.

### Numerical modelling

In this section, we present the results of a numerical study of the propagation of focused cylindrical beams with a phase singularity perpendicular to the axis of a calcite crystal (*CaCO*_3_), having dielectric permittivity *ε*_0_ = 2.232 and *ε*_1_ = 2.376. For comparative modelling of focusing singular beams with cylindrical polarisation of various orders, we use a Gaussian beam as an illuminating beam:

13 $${E}_{0}(r)={r}^{p}\exp \left(-\frac{{r}^{2}}{2{w}^{2}}\right),$$

where *w* is the waist radius Gaussian beam. The following parameters were used in the simulation: laser radiation wavelength *λ* = 532 nm, Gaussian beam waist radius *w* = 100 *μ*m, lens focal length *f* = 7.5 mm.

**Figure 1:**
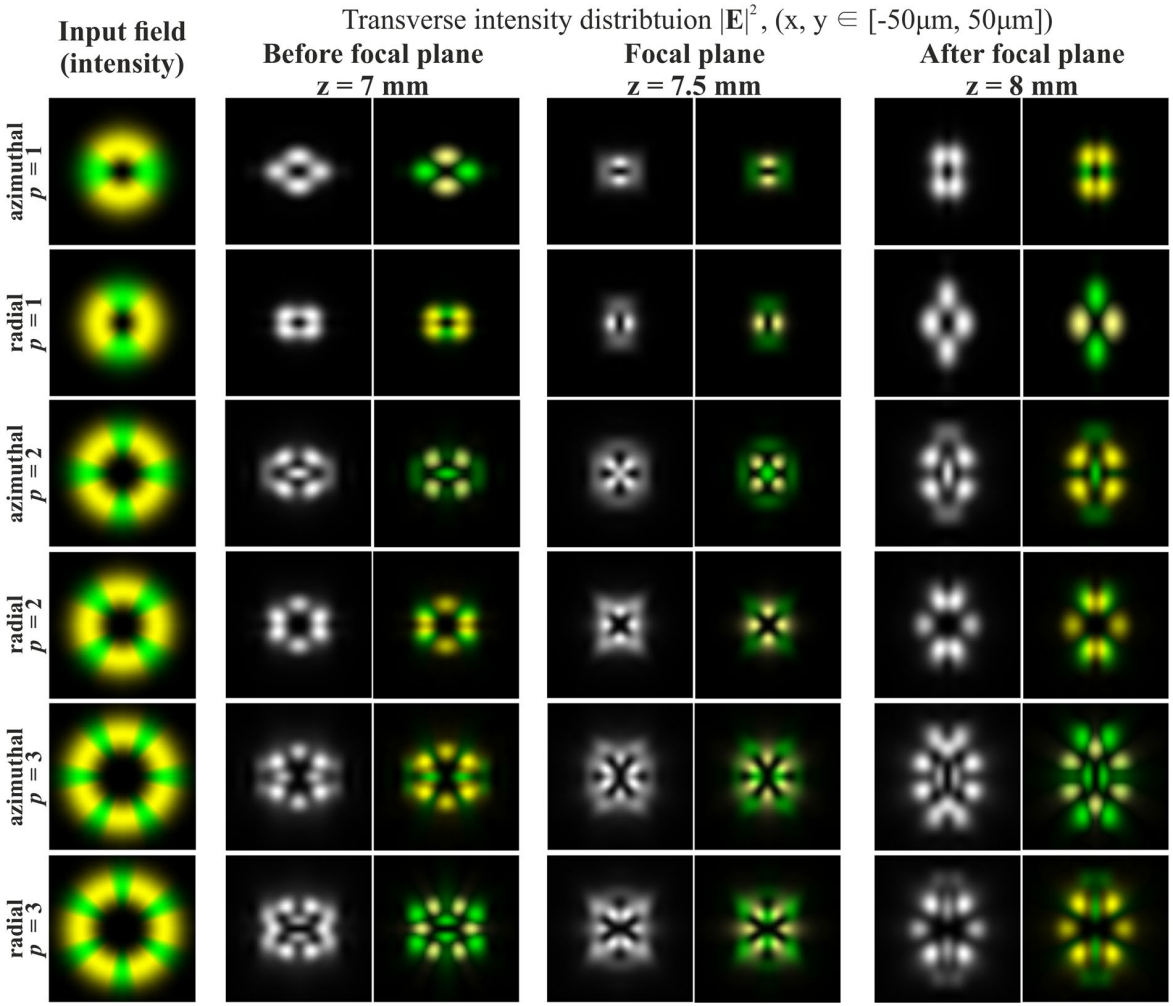
Modelling of the focusing of the Gaussian beam with cylindrical polarisation of various orders *p* in the absence of phase singularity (the crystal axis is directed along the coordinate axis *Y*; yellow colour for the *x*-component, green colour for the *y*-component).

Figure 1 shows the results of the numerical modelling of the focusing of a Gaussian beam with *p*-th-order cylindrical polarisation in the absence of phase singularity. The results clearly show that, as predicted in the theoretical part, the *x*-component of the beams does not change its structure, only transforming on a scale in accordance with the distance from the focal plane. The *y*-component of the beams is subjected to an astigmatic transformation. The overall intensity is a sum of the intensities of the *x*- and *y*-components (the longitudinal component is small and not visually observed) and can be dynamically changed by rotating the polarisation analyser.

At sharp focusing in free space, the radial polarisation (second row of Fig. 1) differs noticeably from azimuthal polarisation (first row of Fig. 1) in the presence of the longitudinal component of the electric field^25,33,36^. However, in the considered case, the results for cylindrical polarisations are similar up to rotation. This is due to the insufficiently-high numerical aperture of the focusing element. The CVBs with an even order of azimuthal polarisation (third row of Fig. 1) have a non-zero intensity value in the centre, associated with the astigmatic transformation of the *y*-component consistent with the even cosine function. For even orders of radial polarisation, this phenomenon is not observed, since the *y*-component is consistent with an odd sine function. To obtain this effect, regardless of the type of polarisation (the dependence on parity of the polarisation order will remain), it is necessary to rotate the crystal around its axis at an angle of 45 degrees.

**Figure 2:**
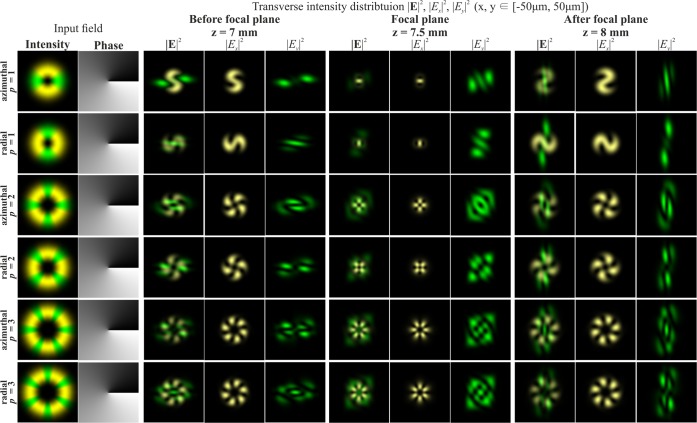
Modelling of the focusing of the Gaussian beam with the first-order vortex phase (*m* = 1) for cylindrical polarisation of various orders *p*.

The introduction of a phase singularity into the initial laser beam additionally complicates the generated intensity patterns. The results of modelling for the Gaussian beam with the first-order vortex phase (*m* = 1) for cylindrical polarisations of various orders *p* are shown in Fig. 2. In this case, a non-zero intensity value at the centre of the beam appears if the parity of the order of polarisation *p* and the vortex number *m* coincide. Moreover, this effect does not depend on the type of polarisation, and rotation of the crystal is not required. Thus, the addition of the vortex phase singularity into the beam makes it possible to reveal more information about the polarisation state of the beam.

Phase singularity for the recognition of the type of polarisation has been used in several works^46–48^, but a confident difference is provided only for uniform and inhomogeneous polarisation^46^. In order to distinguish the first-order radial polarisation from the azimuthal, sharp focusing is necessary^47^. The approach proposed in this paper allows one to visually distinguish the CVBs of different orders, even in the paraxial case. This possibility is required for compressed transmission of information based on polarisation multiplexing of communication channels^29,48^. As seen from Fig. 2, in the paraxial case, the patterns for azimuthal and radial polarisations differ only by a rotation of 90 degrees and a mutual interchanging of the *x*- and *y*-components. Therefore, this paper considers only radial polarisation in what follows.

**Figure 3:**
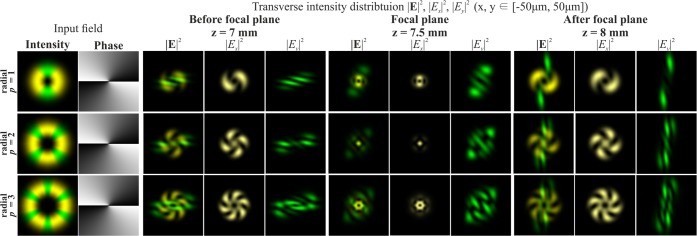
Modelling of the focusing of the Gaussian beam with the second-order vortex phase (*m* = 2) for radial polarization of various orders *p*.

Figure 3 shows the simulation results of focusing the Gaussian beam with the second-order vortex phase (*m* = 2) for radial polarisation of various orders *p*. In this case, a non-zero value also appears in the centre of the beam if the parity of the order of polarisation *p* and the order of the vortex *m* coincide.

**Figure 4:**
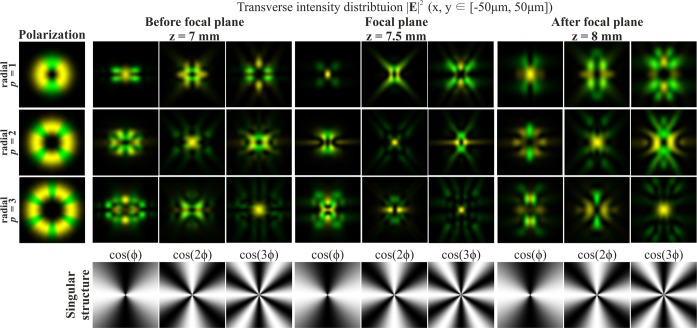
Modelling of the focusing of the Gaussian beam with the singular structure $\cos (m\phi )$ for radial polarization of various orders *p*.

It should be noted that when the order of polarisation p coincides with the order of the optical vortex *m* (the first and second rows of Fig. 2 and the second row of Fig. 3), a bright light spot forms on the optical axis. This effect follows from Eq. (12), since in this case, the sum contains a non-vortex term and, therefore, a non-zero intensity value will be on the optical axis. Illustrations of this effect are also shown in Fig. 4, which demonstrates the modelling results of the focusing of the Gaussian beam with the singular structure, which is the superposition of conjugated optical vortices $\cos (m\phi )=\left[\exp (im\phi )+\exp (-im\phi )\right]$/2, for radial polarisation of various orders *p*.

**Figure 5:**
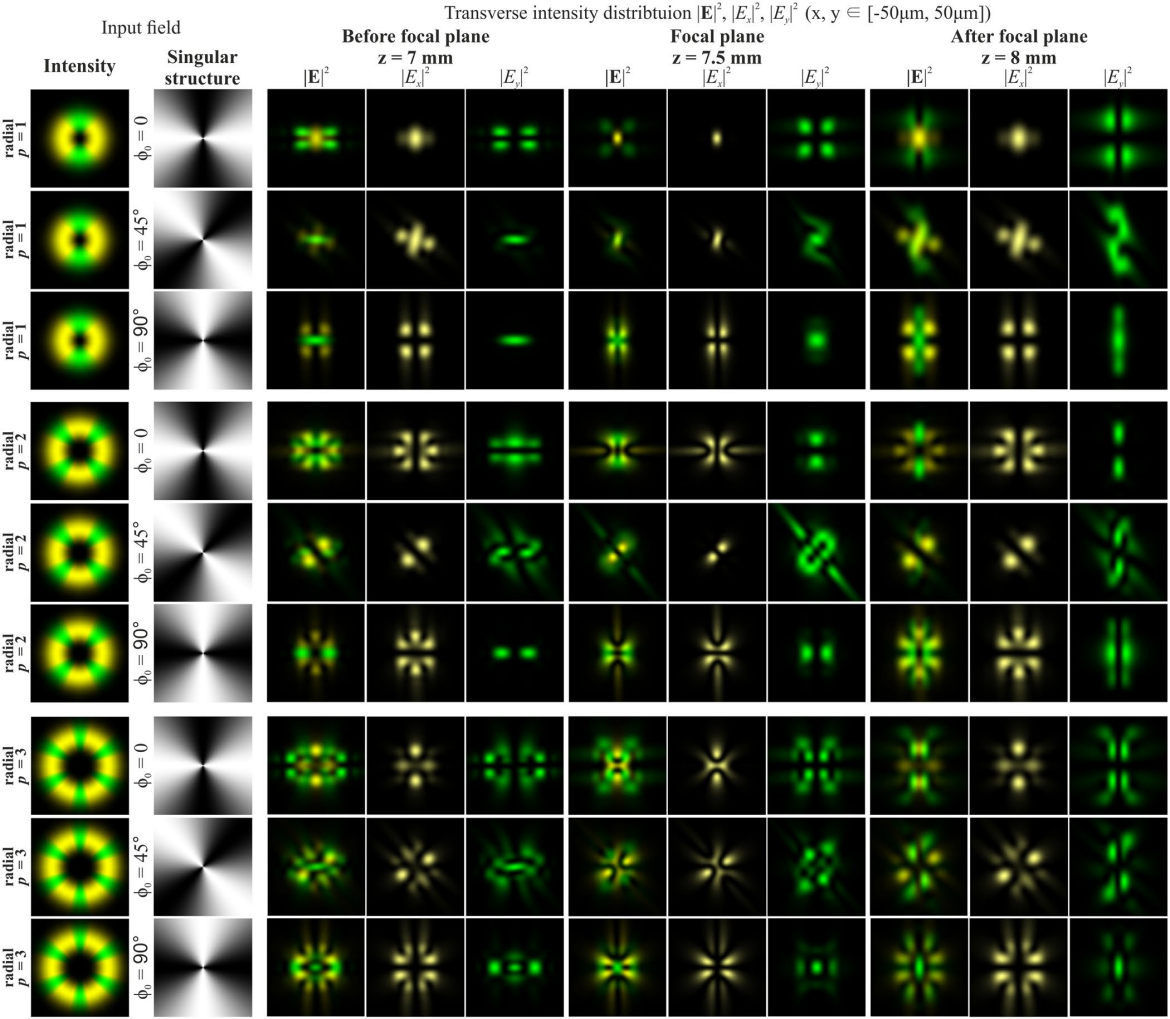
Modelling of the focusing of the Gaussian beam with the singular structure $\cos (\phi +{\phi }_{0})$ for radial polarization of various orders *p*.

**Figure 6:**
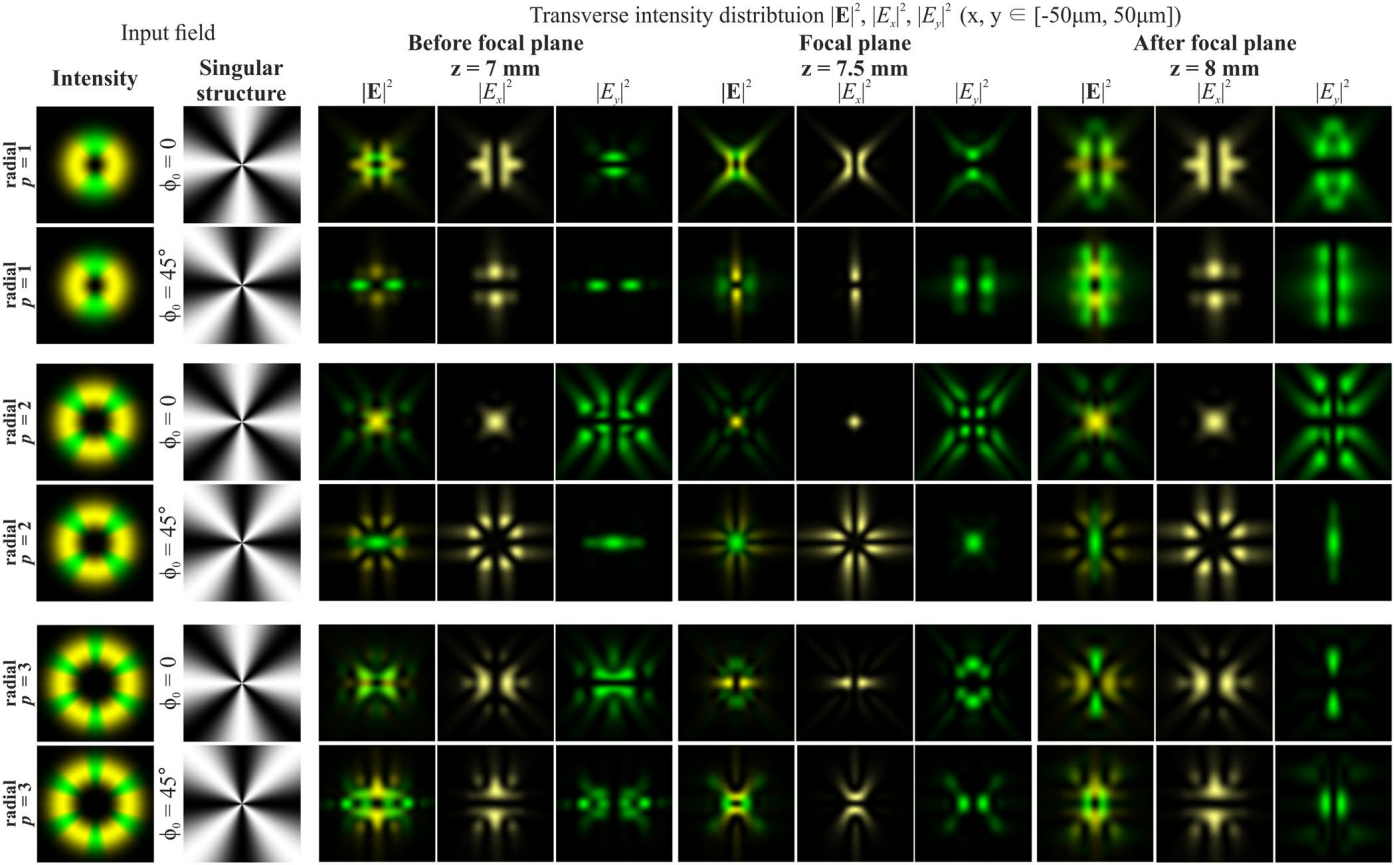
Modelling of the focusing of the Gaussian beam with the singular structure $\cos (2\phi +{\phi }_{0})$ for radial polarization of various orders *p*.

The singularity of the angular cosine (or sine) structure $\cos (m\phi )$ consists of the phase jump and phase uncertainty on the lines where $\cos (m\phi )=0$. Since this structure is not invariant to rotation (similarly to beams with the cylindrical polarisation of orders *p* > 1), additional transformation effects will be observed during rotation of this singular structure. Figures 5 and 6 illustrate this effects for $\cos (\phi +{\phi }_{0})$ and $\cos (2\phi +{\phi }_{0})$, respectively. As can be seen from the results shown in Fig. 5, for the rotation of the singular structure $\cos (\phi )$, significant transformations of the transverse intensity patterns of both field components are observed. Particularly interesting transformations occur when $\cos (\phi )$ is rotated 90 degrees, i.e., when the singular structure becomes an orthogonal distribution $\sin (\phi )$. In this case, the *x*- and *y*-components of the field actually interchange for *p* = 1 (compare the first and third rows in Fig. 5); for *p* = 2, the components rotate 90 degrees (compare the fourth and sixth rows in Fig. 5); for *p* = 3, more complex changes occur, which lead to the appearance of a bright light spot in the centre (compare the seventh and ninth rows in Fig. 5). Similar effects can be observed when the singular structure $\cos (2\phi )$ is rotated 45 degrees, when it is converted into $\sin (2\phi )$ (see Fig. 6). As the results Fig. 6 show, the *y*-component rotates 90 degrees in almost all considered cases when we compare the field before and after the focal plane.

Particularly interesting transformations occur when the order of polarisation coincides with the phase singularity (*p* = *m*). In this case, the transverse field components actually interchange places and in the area of the central spot, polarisation changes to orthogonal (compare the first and third rows in Fig. 5, as well as the third and fourth rows in Fig. 6). The structure $\cos (m\phi )$ has a 2*m*-th order symmetry, and, as *m* increases, even small rotation angles are enough to obtain visually-noticeable changes. To obtain an orthogonal state, it is sufficient to rotate through the angle of 90 degrees per metre.

### Experiments

**Figure 7:**
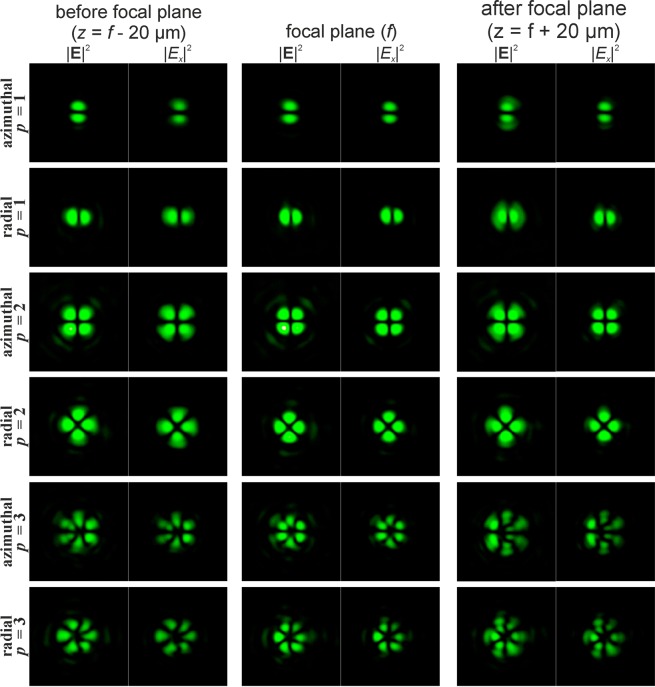
Experimentally obtained intensity distributions of focused CVBs of different orders *p* passed through the biaxial crystal in the absence of phase singularity (the crystal axis is directed along the coordinate axis). The size of images is 350 × 350 *μ*m.

**Figure 8:**
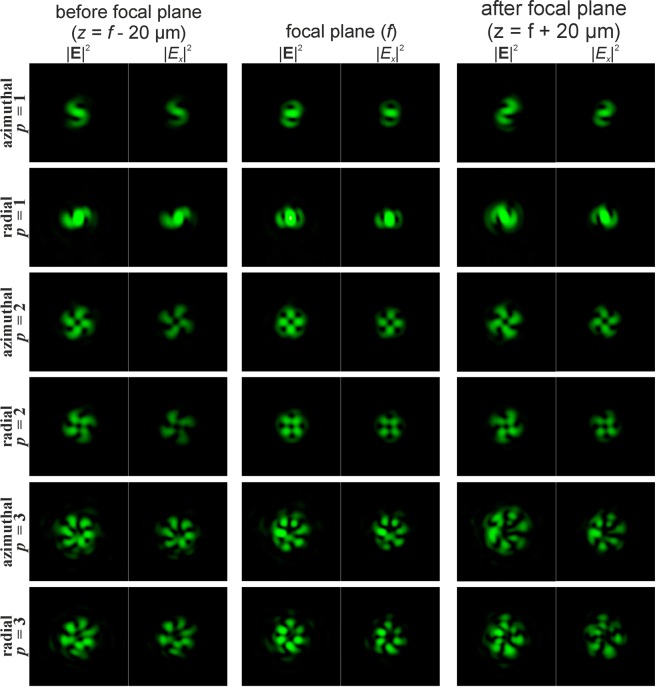
Experimentally obtained intensity distributions of focused CVBs of different orders *p* with the first-order vortex phase (*m* = 1). The size of images is 350 × 350 *μ*m.

**Figure 9:**
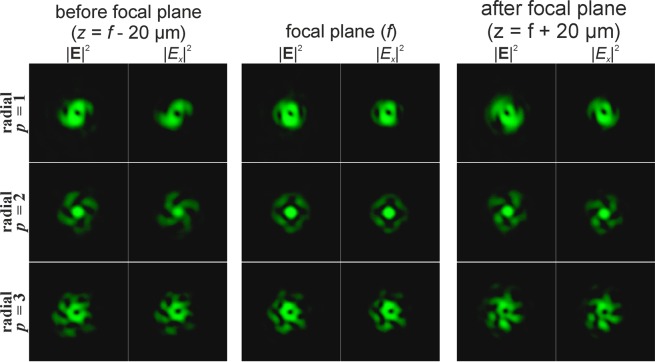
Experimentally obtained intensity distributions of focused radially-polarized beams of different orders *p* with the second-order vortex phase (*m* = 2) passed through the biaxial crystal (the crystal axis is directed along the coordinate axis). The size of images is 350 × 350 *μ*m.

**Figure 10:**
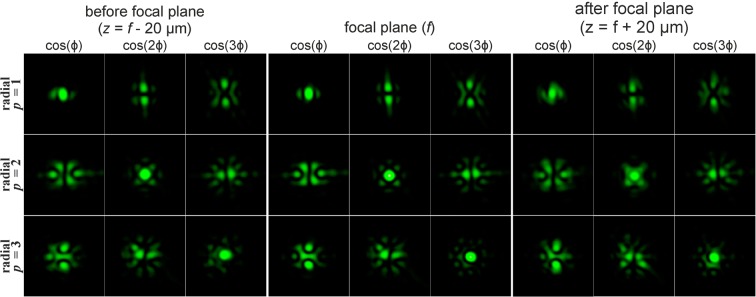
Experimentally obtained intensity distributions of focused Gaussian beams with the singular structure $\cos (m\phi )$ and radial polarization of different orders *p*. The size of images is 350 × 350 *μ*m.

**Figure 11:**
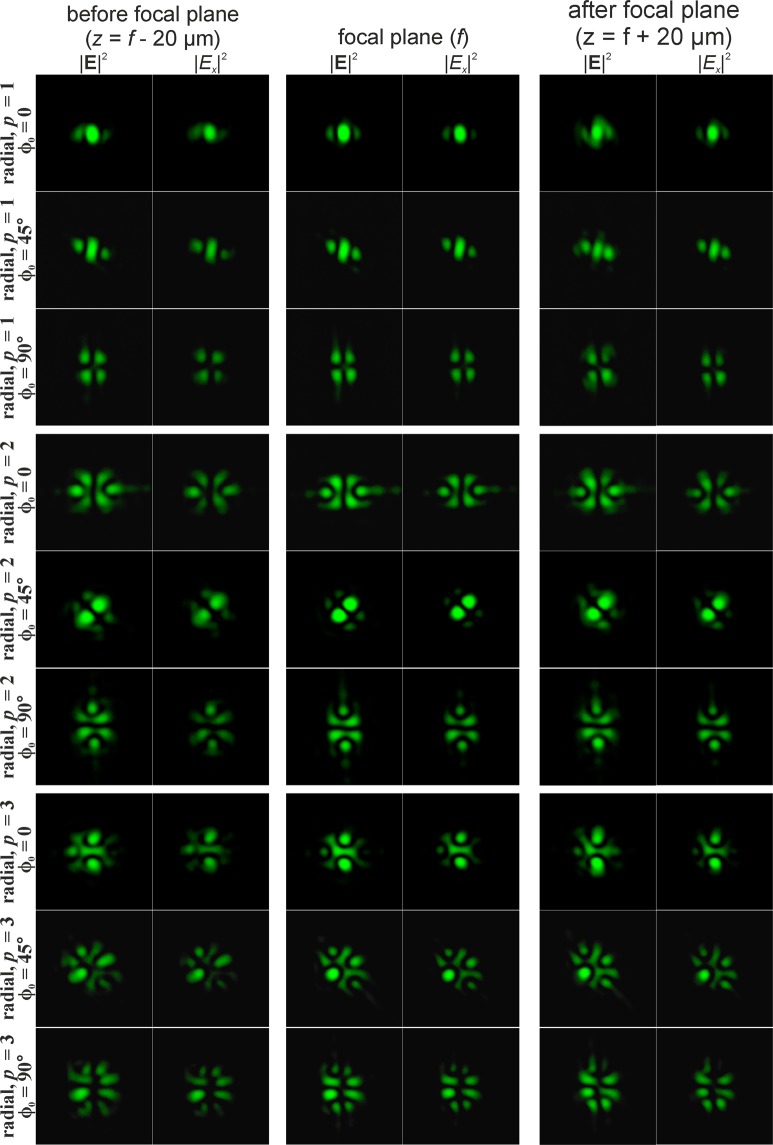
Experimentally obtained intensity distributions of focused Gaussian beams with the singular structure $\cos (\phi +{\phi }_{0})$ and radial polarization of different orders *p* passed through the biaxial crystal (the crystal axis is directed along the coordinate axis). The size of images is 350 × 350 *μ*m.

**Figure 12:**
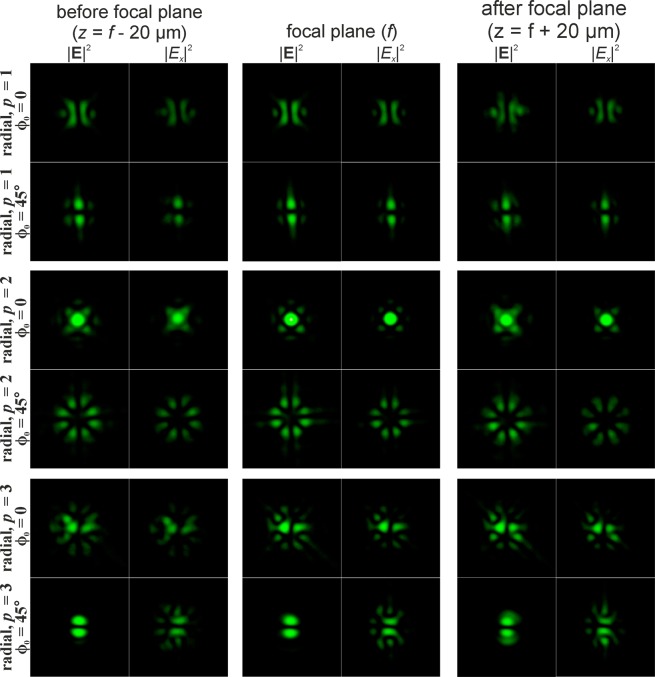
Experimentally obtained intensity distributions of focused Gaussian beams with the singular structure $\cos (2\phi +{\phi }_{0})$ and radial polarization of different orders *p* passed through the biaxial crystal (the crystal axis is directed along the coordinate axis). The size of images is 350 × 350 *μ*m.

The results obtained in experiments for some simulated cases are shown in Figs. 7, 8, 9, 10, 11 and 12. The *x*-components of the experimentally-generated laser beams are in good agreement with modelling results. However, the *CaCO*_3_ crystal used in the experiment was not thick enough to perform a full transformation of the *y*-components of the generated CVBs, and the *y*-components were very low. Because of this, we present only the total intensity distribution and the intensity distribution for the *x*-components of the generated CVBs. The experimentally obtained results confirm the theoretical prediction, mentioned above, that anisotropic crystals can be used not only for determining the polarisation order, but also for a visual detection of the difference between azimuthal and radial polarisation (see Figs. 2 and 8). Slight deviations of the experimental results from the simulation results can be explained by the aberrations of the optical system, thus leading to the splitting of higher-order vortices into low-order vortices with smaller TCs^49,50^, as well as deviations of the profile of the laser beam from the ideal.

## Conclusion and discussion

The presented investigation has shown that, when cylindrically-polarised singular beams are focused perpendicularly to the axis of an anisotropic crystal, a selective (polarisation-dependent) astigmatic transformation of individual components of the electric field occurs. The transverse intensity distribution of the focused field depends not only on the polarisation state of the beam incident on the crystal, but also on the detection plane: the transverse intensity changes significantly before and after the focal plane. The introduction of phase singularities into the initial CVB beam, both as separate optical vortices of different orders and as the result of their superposition, allows one not only to better understand the details of the astigmatic transformation, but also to visually detect the polarisation state of the radiation incident on the crystal. In particular, when the order of polarisation coincides with the order of the optical vortex, a bright light spot forms on the optical axis. In addition, orthogonal polarisation transformations occur in this case.

An additional degree of freedom in analysing the characteristics of a beam incident on a crystal is provided by rotating the crystal around its axis. Analogous results can be obtained by rotating the input beam. In fact, in this case it is possible to regulate the astigmatic transformation of the various components of the electric field. The use of a polarisation analyser at the output of the crystal provides the additional possibility of transforming the intensity pattern, which can help obtain more information about the characteristics of the beam.

The experimentally-obtained intensity distributions for the total fields and their *x*-components agree with theoretical predictions and modelling results. However, the thickness of the *CaCO*_3_ crystal used in the experiments was insufficient for the transformation of the *y*-component of the generated CVBs: the value of the *y*-component was very low and did not affect the total field distribution. In order to increase the transformation, the crystals with a high birefringence can be used; for example, *TiO*_2_ crystal has even more birefringence: Δ*n* = *n*_*e*_ − *n*_*o*_ = (2.978 − 2.668) = 0.310 at wavelength 532 nm (in contrast with Δ*n* = *n*_*e*_ − *n*_*o*_ = (1.488 − 1.663) = 0.175 for the *CaCO*_3_ crystals. However, the *TiO*_2_ is quite expensive. The birefringence of liquid crystals (LCs) is slightly higher than birefringence of *CaCO*_3_ (Δ*n* ≈ 0.2). However, it is more interesting that LC’s birefringence changes (decreases) with increasing temperature, which can provide an additional degree of freedom in the considered transformations. A similar dynamic effect can be achieved using electrically controlled crystals, such as Potassium Dideuterium Phosphate (DKDP)^51^.

When using high-NA focusing, additional effects associated with the amplification of the longitudinal component of the electric field are possible^52^. However, it is rather difficult to implement high-NA focusing inside a crystal, since crystals with high birefringence also usually have high refractive indices (*n*_*o*_ = 1.488, *n*_*e*_ = 1.663 for *CaCO*_3_, *n*_*o*_ = 2.978, *n*_*e*_ = 2.668 for *TiO*_2_). Thus, even if we can achieve high NA = sin*σ* in air (the longitudinal component becomes significant at NA > 0.7), when the radiation passes through the crystal, the angle *σ*_*crystal*_ (at which the rays go out) will decrease in proportion to the refractive index (sin*σ*_*crystal*_ = sin *σ*/*n*_*e*_ ≈ 0.4 according to Snell’s law).

The demonstrated results can be useful for detecting singular fields with different orders of both the vortex phase and cylindrical polarisation in optical communication systems operating on the principle of mode- and polarisation-division multiplexing. In particular, an interesting opportunity to distinguish azimuthal polarisation of the *p*-th order from radial polarisation of the same order was demonstrated. In addition, the CVBs with the complex shape of the intensity distribution hold promise for the realization of efficient optical trapping of nano- and microparticles^53,54^. Thus, it has recently been shown that choosing the right polarisation distribution enhances the trapping efficiency for particles of certain sizes^55^. Such complex CVBs also allow one to improve the axial trapping efficiency for non-spherical nano- and microobjects, similar to the case of trapping of carbon nanotubes^56^. Structured spin angular momentum in highly focused CVBs can also be effectively transferred to the optical torque for the non-magnetic absorptive particle^57^. Moreover, of note here is the demonstrated possibility of the generation of the CVBs with complex shape of the intensity distribution changing after the propagation through the focal plane; indeed, this is useful for the laser processing of bulk transparent materials as well as polymers for fabrication of complex three-dimensional metastructures by single-shot pulse laser printing: the possibility of the use of the structured laser beams for fabrication of chiral three-dimensional metamaterials has recently been demonstrated^58^ and provided a new promising technique for high-throughput laser material processing.

## Methods

**Figure 13:**
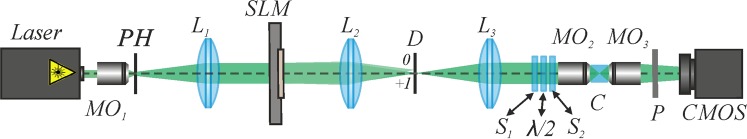
Experimental setup for investigation of transformation of the CVB of the different orders performed by a biaxial crystal. A combination of the first-order *S*-waveplate *S*_1_, the second-order *S*-waveplate *S*_2_ and a *λ*/2-waveplate was used for generation of the CVBs of different orders.

The optical setup for the experimental investigation of the astigmatic transformation of vortex beams performed by a biaxial crystal is shown in Fig. 13. The input laser beam was extended and spatially filtered with a system composed of a microobjective *MO*_1_ (10×, *NA* = 0.2), a pinhole *PH* (aperture size 40 *μ*m), and a lens *L*_1_ (focal length 150 mm). The collimated laser beam was directed onto the display of a transmissive spatial light modulator *SLM* (HOLOEYE, LC 2012 with 1024 × 768 pixel resolution). The SLM was used to realise the phase masks employed in the experiments. A diaphragm (*D*) blocked the zero diffraction order, and a combination of lens *L*_2_ and lens *L*_3_, both with focal lengths of 150 mm, imaged a plane conjugated to the plane of the SLM display in the input pupil of a microobjective *MO*_2_ (16×, *NA* = 0.3). The microobjective *MO*_2_ focused the generated beam inside a cubic biaxial *CaCO*_3_ crystal *C* (8 × 8 × 8 mm), and a microobjective *MO*_3_ (16×, *NA* = 0.3) imaged the transformed optical field distributions onto the sensor of a CMOS-video camera. A polariser *P* was used as an analyser of light polarisation. To generate the first, second, and third-order CVBs, we used commercially available high-quality *S*-waveplates of first and second order (also called zero-order vortex half-wave plates, Thorlabs Inc.) and their combinations with a *λ*/2 waveplate^59^.
